# Supplementary material for: Identification of photoperiod-induced specific miRNAs in the adrenal glands of Sunite sheep (Ovis aries)
Source: Front Vet Sci. 2022 Jul 22;9:888207. doi: 10.3389/fvets.2022.888207 (PMC9354845; doi:10.3389/fvets.2022.888207)
Supplement: Supplementary file 1 [file Data_Sheet_1.ZIP › Supplementary file/miRDeep2 file.zip!/miRDeep2 file/Know_Structure/LP42A2/oar-mir-154b.pdf]

miRBase precursor : oar-mir-154b
Total read count : 1367
oar-miR-154b-5p read count : 1265
oar-miR-154b-3p read count : 96
remaining reads : 6

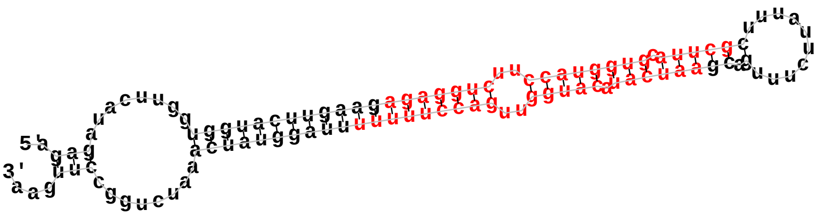

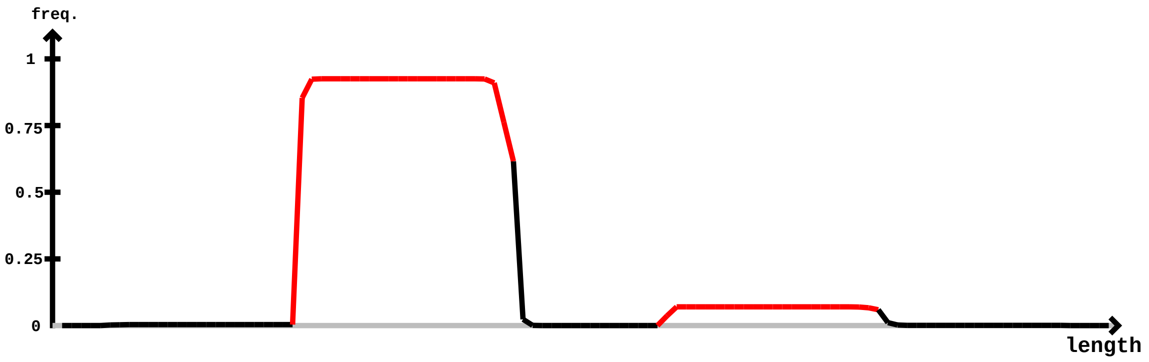

oar-miR-154b-3p

oar-miR-154b-5p

5'- agagauacuuggugguacuugaagagaggucuuccauggugcauucgcuuuauucuuugacgaaucauacaugguugaccuuuuuuuagguaucaaaucuggccuugaa -3'	exp		
.(((........(((((((((((((((((((..((((((((.((((((..........).)))))))).)))))..)))))))))))))))))))........)))...	reads	mm	sample
....auacuuggugguacuugaag...	3	0	seq
.....uacuuggugguacuugaag...	1	0	seq
......acuuggugguacuugaag...	1	0	seq
........................agaggucuuccauggugca..	1	0	seq
........................agaggucuuccauggugcau...	20	0	seq
........................agaggucuuccauggugcauu..	191	0	seq
........................agaggucuuccauggugcauuc...	192	0	seq
........................agaggucuuccauggugcauucg..	737	0	seq
........................agaggucuuccauggugcauucgc...	25	0	seq
........................agaggucuuccauggugcauucgcu..	1	0	seq
.........................gaggucuuccauggugcauu..	10	0	seq
.........................gaggucuuccauggugcauuc...	9	0	seq
.........................gaggucuuccauggugcauucg..	71	0	seq
.........................gaggucuuccauggugcauucgc...	6	0	seq
..........................aggucuuccauggugcauucg..	2	0	seq
....aaucauacaugguugaccuu...........................	1	0	seq
....aaucauacaugguugaccuuu..........................	4	0	seq
....aaucauacaugguugaccuuuu.........................	8	0	seq
....aaucauacaugguugaccuuuuu........................	34	0	seq
....aaucauacaugguugaccuuuuuu.......................	3	0	seq
....aucauacaugguugaccuuuu.........................	2	0	seq
....aucauacaugguugaccuuuuu........................	33	0	seq
....aucauacaugguugaccuuuuuu.......................	9	0	seq
....aucauacaugguugaccuuuuuuu......................	2	0	seq
....uuagguaucaaaucuggcc.....	1	0	seq
